# Microgliosis in the Spinal Dorsal Horn Early After Peripheral Nerve Injury Is Associated with Damage to Primary Afferent Aβ-Fibers

**DOI:** 10.3390/cells14090666

**Published:** 2025-05-02

**Authors:** Yuto Shibata, Yuki Matsumoto, Keita Kohno, Yasuharu Nakashima, Makoto Tsuda

**Affiliations:** 1 Department of Molecular and System Pharmacology, Graduate School of Pharmaceutical Sciences, Kyushu University, 3-1-1 Maidashi, Higashi-ku, Fukuoka 812-8582, Japan; 2 Department of Orthopaedic Surgery, Graduate School of Medical Sciences, Kyushu University, 3-1-1 Maidashi, Higashi-ku, Fukuoka 812-8582, Japan; 3 Kyushu University Institute for Advanced Study, 744 Motooka Nishi-ku, Fukuoka 819-0395, Japan

**Keywords:** microglia, primary afferents, Aβ-fibers, C-fibers, peripheral nerve injury, spinal dorsal horn

## Abstract

Neuropathic pain results from a lesion or disease affecting the somatosensory nervous system. Injury to primary afferent nerves leads to microgliosis in the spinal dorsal horn (SDH), which plays a crucial role in developing neuropathic pain. Within the SDH, primary afferent fibers broadly project, and microglia are nearly ubiquitously distributed under normal conditions. However, not all microglia react to injuries affecting primary afferent fibers, resulting in spatially heterogeneous microgliosis within the SDH. The mechanisms underlying this phenomenon remain elusive. In this study, the spatial relationship between microgliosis and the projections of injured nerves was investigated by generating mice that had expressed tdTomato in the fourth lumbar dorsal root ganglion (L4-DRG) neurons via intra-L4-spinal nerve (SpN) injection of adeno-associated viral vectors. After transection of the L4-SpN, we found that microgliosis in the SDH selectively occurred in the innervation territories of the injured primary afferent fibers. However, denervating transient receptor potential vanilloid 1 (TRPV1)-expressing primary afferent fibers in the SDH through intrathecal injection of capsaicin did not trigger microgliosis, nor did it influence the microgliosis induced by L4-SpN injury. Conversely, pharmacological damage to myelinated DRG neurons, including Aβ-fibers, was sufficient to induce microgliosis. Furthermore, L4-SpN injury also induced microgliosis in the gracile nucleus, which primarily receives innervation from Aβ-fibers. These findings suggest that microgliosis in the SDH shortly after peripheral nerve injury is predominantly associated with damage to primary afferent Aβ-fibers.

## 1. Introduction

Neuropathic pain arises from lesions or diseases of the somatosensory nervous system. Increasing evidence from studies on neuropathic pain models shows that nerve damage causes significant changes not only in neurons [1,2,3] but also in non-neuronal cells [4,5], particularly microglia (tissue-resident macrophages), in the central nervous system (CNS). Following peripheral nerve damage, microglia in the spinal dorsal horn (SDH) respond rapidly, undergoing extensive changes in morphology, cell number, marker expression, transcriptional and translational activities, and function [4,6]. These reactive microglia in the SDH, evident shortly after nerve damage, play a critical role in the subsequent pathological changes in CNS function and the development of neuropathic pain [4,6]. Therefore, understanding the reactive processes of microglia triggered by nerve damage is crucial for elucidating the onset of neuropathic pain and developing therapeutic strategies.

Nerve damage-induced reactive microglia have been widely documented across various experimental models. A traditional model involves transection of peripheral nerves, such as the sciatic nerve, which leads to molecular and cellular changes in microglia within the spinal cord (both ventral and dorsal horns) [4,7,8,9]. Such alterations commonly follow various types of injuries (e.g., partial/complete ligation, or compression) to different peripheral nerve sites [sciatic nerve, tibial nerve, common peroneal nerve, and spinal nerve (SpN)] [4,10,11,12,13,14]. Unlike peripheral tissue inflammation, nerve injury clearly induces molecular and cellular changes in the microglia of the SDH [8,13,15,16]. Damage to primary afferent fibers plays a critical role in these reactive processes. In the SDH, primary afferent fibers are widely innervated, and under normal conditions, microglia are almost ubiquitously distributed. Interestingly, not all microglia respond to injuries affecting primary afferent nerves, leading to spatial heterogeneity in reactive microglia within the SDH [17]. In particular, areas in the SDH where microglia exhibit morphological changes and high expression of ionized calcium-binding adapter molecule 1 (IBA1, a microglial marker) are preferentially observed [18]. However, the mechanisms underlying this spatial heterogeneity of microglial responses in the SDH following nerve injury remain unclear.

The aim of this study was to investigate this unsolved issue and also to provide a clue to elucidate the role of injured neurons in microglial responses in the SDH and its molecular mechanisms. In this study, the spatial correlation between reactive microglia and injured nerve fiber projections in the SDH was analyzed. For this analysis, a genetic technique was developed to specifically label injured nerve fibers using adeno-associated viral (AAV) vectors to express fluorescent proteins in certain segments of dorsal root ganglion (DRG) neurons, specifically the fourth lumbar (L4). Using mice with labeled L4-DRG and pharmacological methods to damage either myelinated primary afferent fibers (particularly Aβ-fibers) or unmyelinated nociceptors, this study showed that the spatial heterogeneity of microglial responses [upregulation of IBA1 expression and an increase in microglial cell numbers (referred to as microgliosis in this study)] within the SDH following nerve injury correlates with the projection territories of damaged primary afferent Aβ-fibers.

## 2. Materials and Methods

### 2.1. Animals

Male C57BL/6J mice (CLEA Japan, Tokyo Japan) and male B6.Cg-*Gt(ROSA)26Sor^tm14(CAG-tdTomato)Hze^*/J (*ROSA26*^tdTomato^) mice (Stock No: 007914, The Jackson Laboratory, Bar Harbor, ME, USA) were used. All mice used were aged 8–10 weeks at the start of each experiment and were housed individually or in groups at a temperature of 22 ± 1 °C with a 12 h light–dark cycle, and were fed food and water ad libitum. All animal experiments were conducted according to relevant national and international guidelines contained in the “Act on Welfare and Management of Animals” (Ministry of Environment of Japan) and the “Regulation of Laboratory Animals” (Kyushu University) and under the protocols approved by the Institutional Animal Care and Use committee review panels at Kyushu University.

### 2.2. Recombinant AAV Vector Production and L4-SpN Injection

Viral vector production was performed according to our previous method [19]. The gene-encoding Cre and tdTomato were subcloned into the pENTR plasmid (Thermo Fisher Scientific, Waltham, MA, USA). To produce AAV vectors, Cre and tdTomato were inserted into pZac2.1-enhanced synapsin promoter (ESYN)-WPRE. rAAV vectors were produced from human embryonic kidney 293T (HEK293T) cells with triple transfection [pZac, cis plasmid; pAAV2/9 (University of Pennsylvania Gene Therapy Program Vector Core), trans plasmid; pAd DeltaF6, adenoviral helper plasmid (University of Pennsylvania Geno Therapy Program Vector Core)] and purified by two cesium chloride density gradient purification steps. The vector was dialyzed against phosphate-buffered saline (PBS) containing 0.001% (*v*/*v*) Pluronic-F68 (Thermo Fisher Scientific, Waltham, MA, USA) or 0.001% (*v*/*v*) poloxamer 188 Non-ionic Surfactant (#24040032; Thermo Fisher Scientific, Waltham, MA, USA) using Vivaspin Turbo 15 100,000 MWCO (#VS15T41; Sartorius, Gottingen, Germany). The genome titer of rAAV was determined by Pico Green fluorometric reagent (#P7589; Thermo Fisher Scientific, Waltham, MA, USA) following denaturation of the AAV particles. Vectors were stored at −80 °C until use.

Viral injections were performed according to our previously described method [19]. Mice were deeply anesthetized via intraperitoneal (i.p.) injection using a mixture of anesthetic agents: medetomidine hydrochloride (0.15 mg/kg), midazolam (2 mg/kg), and butorphanol tartrate (2.5 mg/kg). The skin was incised at L3–S1, and the paraspinal muscles and fat were removed to expose the L5-traverse process, revealing the parallel-lying L3- and L4-SpNs. A glass microcapillary, filled with rAAV solution (AAV-ESYN-tdTomato, or AAV-ESYN-Cre), was inserted into the L4-SpN to a depth of 150 μm from the surface of the nerve. Microinjection of 200 nL of rAAV solution into wild-type (WT) and *ROSA26*^tdTomato^ mice, respectively, was performed using a Micro4 Micro Syringe Pump Controller (World Precision Instruments, Sarasota, FL, USA). After microinjection, the glass microcapillary was removed, the skin was sutured with 5-0 silk, and the mice were kept under a heating light until they recovered. According to our previous method [19], the virus-injected mice were used for further analyses 3 weeks after injection. The viral titers used were AAV2/9-ESYN-tdTomato and AAV2/9-ESYN-Cre, both at 1.0 × 10^13^ GC/mL.

WT mice were injected with saporins into the L4-SpN using a Micro4 Micro Syringe Pump Controller, administering 400 nL of saporin solution [saporin conjugated with cholera toxin B subunit (CTB-SAP; IT-14, Advanced Targeting Systems, Carlsbad, CA, USA), isolectin B4 (IB4-SAP; IT-10, Advanced Targeting Systems, Carlsbad, CA, USA), and unconjugated saporin (Ctrl-SAP; PR-01, Advanced Targeting Systems, Carlsbad, CA, USA)] and PBS as controls. CTB-SAP [20] and IB4-SAP [21] are known to be internalized by myelinated and unmyelinated afferents, respectively, and induce neuronal death through ribosomal inactivation. Fourteen days after injection, the presence of IBA^+^ microglia in the L3/4-SDH and neurofilament 200 (NF200)^+^ or IB4^+^ neurons in the L4-DRG was analyzed by immunohistochemistry. To achieve a dose of 0.6 pmol/400 nL/mouse, saporin concentrations were adjusted in PBS as follows: Ctrl-SAP, 0.05 mg/mL; CTB-SAP, 0.2 mg/mL; IB4-SAP, 0.3 mg/mL.

### 2.3. Peripheral Nerve Injury

An SpN injury model was used [22] with some modifications, as described previously [23]. Briefly, under isoflurane (2%) anesthesia, a small incision at L3–S1 was made. The paraspinal muscle and fat were removed from the L5 traverse process, which exposed the parallel-lying L3- and L4-SpNs. The L4-SpN was then carefully isolated and cut. The wound and the surrounding skin were sutured with 5-0 silk. In experiments of a combination with AAV microinjection, the injection site was exposed again, and the L4-SpN was carefully cut.

### 2.4. Hot Plate Test

To assess thermosensory behaviors, mice were placed on a metal surface (25 × 20 cm) maintained at 55 °C within a 25 cm-high Plexiglass box. The latency to either lick the hind paw or jump was recorded as a nocifensive end point [24].

### 2.5. Intrathecal Injection

Under 2% isoflurane anesthesia, a 30 G needle attached to a 25 μL Hamilton syringe was inserted into the intervertebral space between the L5 and L6 spinal vertebrae in mice, as previously described [23,25]. To ablate transient receptor potential vanilloid 1 (TRPV1^+^) afferent fibers in the SDH, WT mice were injected intrathecally with capsaicin [10 μg/5 μL; #M2028, Sigma, Saint Louis, MO, USA; capsaicin was first dissolved in ethanol and diluted by PBS with Tween-80 (the final concentration of ethanol and Tween-80 was 10%)] [26] or vehicle (10% ethanol/10% Tween 80/PBS). Denervation of TRPV1^+^ nerve fibers in the SDH was assessed 3 and 7 days after capsaicin injection by immunostaining. Microglial response in the SDH was observed 7 days after L4-SpN injury was performed at 7 days post-capsaicin treatment.

### 2.6. Immunohistochemistry

Mice were deeply anesthetized with i.p. injection of pentobarbital and transcardially perfused with PBS followed by ice-cold 4% paraformaldehyde/PBS. The transverse L4 segments of the spinal cord were removed and postfixed in the same fixative for 3 h at 4 °C. According to our previous method [23], DRG sections (15 μm) and transverse spinal sections (30 μm) were incubated for 48 h at 4 °C with primary antibodies for anti-glial fibrillary acidic protein (GFAP) (rabbit monoclonal; 1:2000; 13-0300, Invitrogen, Waltham, MA, USA), anti-IBA1 (guinea pig polyclonal; 1:2000; 234 00, Synaptic Systems, Goettingen, Germany), Alexa Fluor 647-conjugated anti- myelin basic protein (MBP) (1:1000; SMI99, BioLegend, San Diego, CA, USA), anti-NF200 (rabbit polyclonal; 1:2000; N4142, Sigma-Aldrich, Saint Louis, MO, USA), biotin-conjugated IB4 (1:1000; I21414, Invitrogen), and anti-TRPV1 (guinea pig polyclonal; 1:1000; GP14100, Neuromics, Waltham, MA, USA). Tissue sections were incubated with secondary antibodies conjugated to Alexa Fluor 488 (1:1000; A-21208, Thermo Fisher, Waltham, MA, USA), Alexa Fluor 546 (1:1000; A-11056, Thermo Fisher, Waltham, MA, USA), or streptavidin Alexa Fluor 488 (1:1000; S11223, Invitrogen, Waltham, MA, USA). For Nissl staining, DRG sections were stained for 30 min at room temperature. The samples were mounted with Vectashield hardmount (Vector Laboratories, Newark, CA, USA) or ProLong Glass Antifade Mountant (Invitrogen, Waltham, MA, USA). Three to five sections from one tissue were randomly selected, and images were taken using a confocal laser microscope imaging system (LSM700/900, Carl Zeiss, Oberkochen, Germany) and analyzed using Image J. The number of tdTomato^+^ cells and NF200^+^ and IB4^+^ cells was counted manually using the cell counter plugin of Image J [19]. For quantification of the number of microglia in the SDH, IBA1^+^ cells with clear cell bodies and with an S/N ratio of 2.0 or more were counted [18]. The numbers of particles was counted using the Analyze Particles function of Image J. The region of interest (ROI) was determined by drawing the boundary between gray and white matter of the SDH based on the differential interference contrast (DIC) image.

### 2.7. Statistical Analysis

Quantitative data are shown as mean ± SEM. Statistical significance was determined using the unpaired *t*-test and one-way analysis of variance (ANOVA) with the post hoc Tukey’s multiple-comparisons test using Prism 7 (GraphPad, San Diego, CA, USA). Values were considered significantly different at *p* < 0.05.

## 3. Results

### 3.1. New Methods to Visualize Injured Primary Afferent Fibers in the SDH Using AAV Vectors

**Figure 1:**
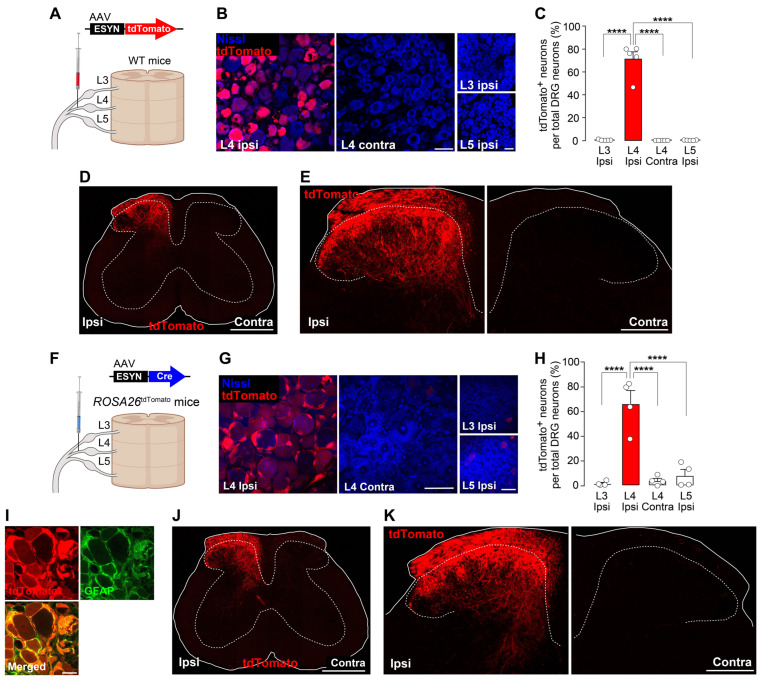
Specific labeling of L4-DRG neurons and their projections in the SDH by injecting AAV vectors into the L4-SpN. (**A**) Schematic illustration of L4-SpN microinjection of AAV-ESYN-tdTomato in WT mice. (**B**) Representative fluorescence images of tdTomato^+^ cells (red) and DRG neurons stained by Nissl (blue) in the L3/4/5 segments. Scale bars, 50 μm. (**C**) Percentage of tdTomato^+^ cells in total Nissl^+^ DRG neurons in each segment [*n* = 5 mice (3 or 4 slices per mouse)]. **** *p* < 0.0001, one-way ANOVA with post hoc Tukey’s multiple-comparisons test. (**D**,**E**) tdTomato expression in the L4-SDH at 3 weeks (**D**) and 4 weeks (**E**) after microinjection of AAV-ESYN-tdTomato. Scale bars; 500 μm (**D**) and 200 μm (**E**). (**F**) Schematic illustration of L4-SpN injection of AAV-ESYN-Cre in *ROSA26*^tdTomato^ mice. (**G**) Representative fluorescence images of tdTomato^+^ cells (red) and Nissl^+^ DRG neurons (blue). Scale bars, 50 μm. (**H**) Percentage of tdTomato^+^ Nissl^+^ neurons per total Nissl^+^ DRG neurons in each segment [*n* = 4 mice tested (4 slices per mouse)]. **** *p* < 0.0001, one-way ANOVA with post hoc Tukey’s multiple-comparisons test. (**I**) Immunolabeling of tdTomato^+^ cells (red) with GFAP (green), a satellite glial marker, in the L4-DRG. Scale bars, 20 μm. (**J**,**K**) tdTomato^+^ nerve fibers in the L4-SDH at 3 weeks (**J**) and 4 weeks (**K**) after microinjection of AAV-ESYN-Cre. Scale bars, 500 μm (**J**) and 200 μm (**K**). Data are shown as the mean ± SEM. Panels A and F were created with BioRender.com.

Initially, the spatial relationship between reactive microglia and injured nerve fiber projections in the SDH following nerve injury were examined. To visualize injured primary afferent fibers, a method for specifically and genetically visualizing neurons in the L4-DRG and their nerve fiber projections was developed using an AAV vector expressing the fluorescent protein tdTomato under the control of the ESYN promoter (AAV-ESYN-tdTomato) (Figure 1A). This AAV was unilaterally injected into the L4-SpN of WT mice. Four weeks later, tdTomato fluorescence was specifically expressed in neurons in the L4-DRG ipsilateral to the injection but not contralateral to it (Figure 1B,C). Additionally, tdTomato was not expressed in the neighboring L3 or L5-DRGs. In the L4-DRG, approximately 70% of the total neurons were positive for tdTomato (Figure 1C). In the spinal cord of the WT mice injected with AAV-ESYN-tdTomato, the dorsal root of the L4-DRG neurons at its entry zone and their nerve fibers in the parenchyma of the SDH were clearly visualized (Figure 1D,E). tdTomato expression was not observed in ventral horn neurons (Figure 1D), confirming the specific labeling of L4-DRG neurons. As a complementary approach, *ROSA26*^tdTomato^ mice in which AAV-ESYN-Cre had been injected into the L4-SpN to induce tdTomato expression were used (Figure 1F). As anticipated, this strategy resulted in expression of tdTomato in L4-DRG neurons (Figure 1G,H); however, unexpectedly, tdTomato expression was also observed in satellite glial cells surrounding the DRG neurons (Figure 1I). Nevertheless, tdTomato^+^ fibers of L4-DRG neurons in the SDH were also clearly observed (Figure 1J,K). Thus, we established two methods for genetically and specifically visualizing L4-DRG nerve fiber projections in the SDH.

### 3.2. Spatial Correlation Between Injured Nerve Fiber Projection and Reactive Microglia

**Figure 2:**
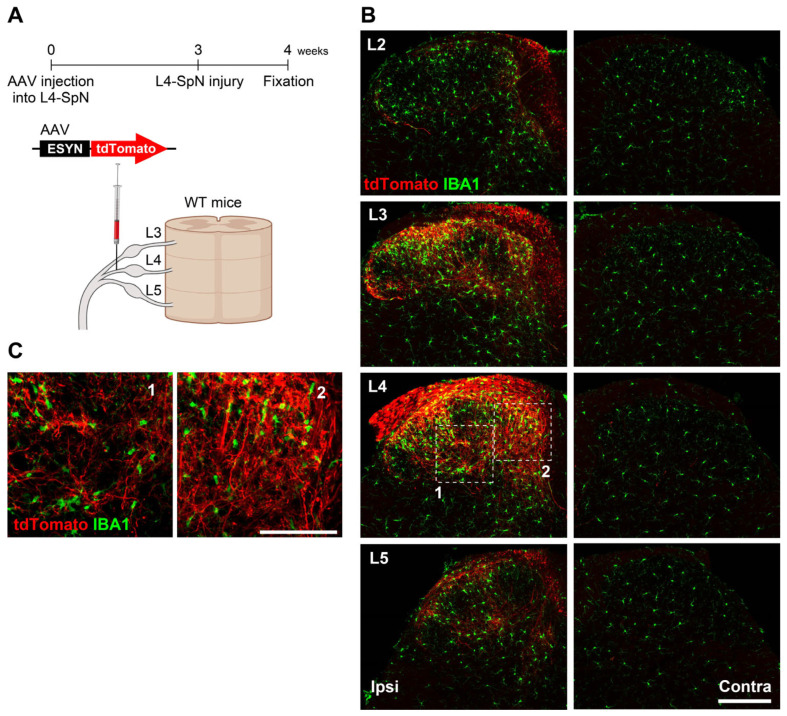
Similarity in spatial patterns of injured L4-DRG spinal nerve projection and reactive microglia in the SDH. (**A**) Schematic timeline and illustration of AAV injection, L4-SpN injury, and fixation. (**B**,**C**) Immunolabeling of IBA1^+^ cells (green) and tdTomato^+^ nerve fibers (red) in each SDH segment and two selected areas (1 and 2) 7 days after L4-SpN injury. Scale bars, 200 µm (**B**) and 100 µm (**C**). Panel A was created with BioRender.com.

Using the established methods, we explored the relationship between the projection territories of injured L4-DRG nerve fibers and the areas of reactive microglia following L4-SpN transection (Figure 2A). L4-SpN transection markedly increased the immunofluorescence levels of IBA1 and the number of microglia (referred to as microgliosis) 7 days post-injury (Figure 2B). Notably, the L4-SpN injury-induced microgliosis was not spatially uniform within the L4-SDH but was selectively observed in the innervation territories of tdTomato^+^ injured L4-DRG nerve fibers (Figure 2B,C). Additionally, consistent with the rostro-caudal projection pattern of L4-DRG neurons in the SDH, the nerve injury-induced microgliosis was observed in the neighboring segments (L2, L3, and L5) (Figure 2B). This spatial pattern also correlated with the projection territories of tdTomato^+^ fibers. These findings provide compelling evidence that microgliosis in the SDH after nerve injury is localized to the projection territories of injured primary afferent nerve fibers.

### 3.3. TRPV1^+^ Primary Afferent Fibers Are Dispensable for Microgliosis in the SDH After Peripheral Nerve Injury

**Figure 3:**
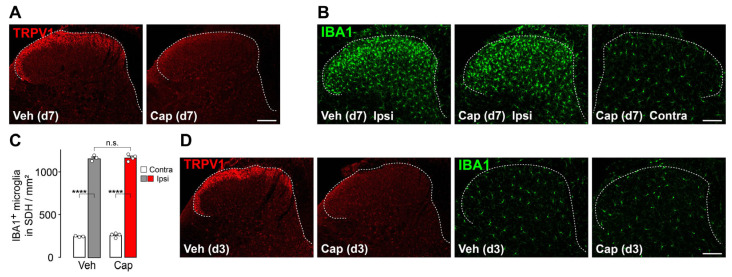
Minimal contribution of TRPV1^+^ primary afferent fibers in the SDH to microgliosis after L4-SpN injury. (**A**) Representative immunofluorescence images of TRPV1^+^ nerve fibers (red) in the SDH 7 days after intrathecal injection of vehicle (Veh) or capsaicin (Cap; 10 μg) to WT mice. Scale bar, 100 μm. (**B**) Representative images of IBA1^+^ cells (green) in the SDH 7 days after L4-SpN injury in WT mice with intrathecal injection of vehicle or capsaicin. Scale bar, 100 μm. (**C**) Quantification of IBA1^+^ cells in the SDH ipsilateral and contralateral to the injury (*n* = 3 to 4 mice). **** *p*  <  0.0001, unpaired *t*-test. n.s., not significant. (**D**) Representative immunofluorescence images of TRPV1^+^ nerve fibers (red) and IBA1^+^ cells (green) in the SDH 3 days after intrathecal capsaicin injection. Scale bar, 100 μm. Data are shown as mean  ±  SEM.

DRG neurons are divided into several classes, such as myelinated and unmyelinated, the latter of which are positive for TRPV1 and IB4 in mice [27]. Their spatial projection patterns in the SDH also differ [27,28,29]. To assess the impact of damage to TRPV1^+^ primary afferent fibers on microglial reactivity in the SDH, mice were treated with an intrathecal injection of capsaicin (10 μg/5 μL), a dose that was sufficient to denervate TRPV1^+^ fibers in the SDH [26]. Seven days post-injection, TRPV1 immunofluorescence in primary afferent fibers in the SDH was absent (Figure 3A), and nocifensive behaviors in response to noxious heat were not observed (the latency to display nocifensive behaviors: vehicle-treated mice, 11.0 ± 0.4 s; capsaicin-treated mice, 30 ± 0.0 s, *n* = 3–4), confirming the denervation of TRPV1^+^ primary afferent fibers in the SDH. In these mice, ablation of spinal TRPV1^+^ nerve fibers had no effect on the L4-SpN transection-induced microgliosis (increased IBA1 immunofluorescence levels and microglial cell count) in the L4-SDH 7 days post-injury (Figure 3B,C). Moreover, at an early time point (3 days post-capsaicin injection), spinal TRPV1^+^ nerve fiber ablation alone did not increase IBA1 immunofluorescence or microglial cell number (Figure 3D). These results indicate that TRPV1^+^ primary afferent fibers are dispensable for microgliosis in response to peripheral nerve injury.

### 3.4. Microgliosis in the SDH Involves Damage to Primary Afferent Aβ-Fibers

**Figure 4:**
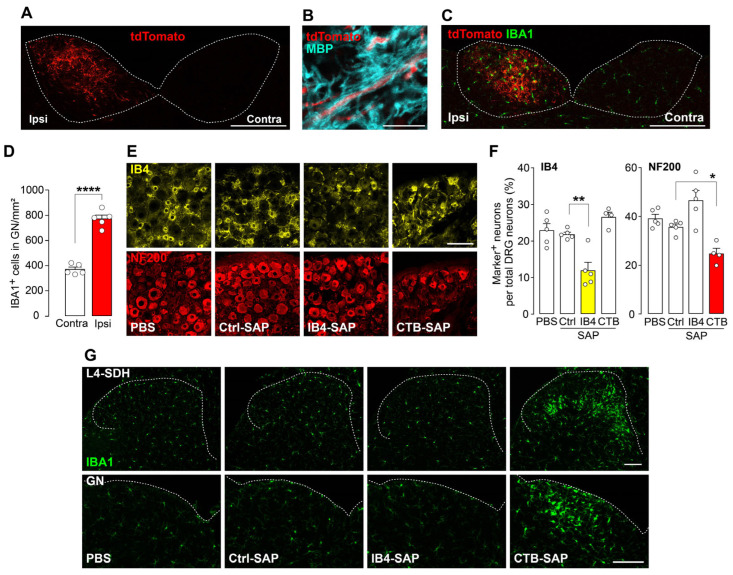
Damage to primary afferent Aβ-fibers is important for L4-SpN injury-induced microgliosis. (**A**) tdTomato expression (red) of primary afferent nerve fibers in the GN. Scale bar, 200 μm. (**B**) Immunostaining of tdTomato^+^ nerve fibers (red) and MBP (cyan) in the GN. Scale bar, 10 μm. (**C**) tdTomato^+^ nerve fibers (red) and IBA1^+^ cells (green) in the GN of WT mice 7 days after L4-SpN injury. Scale bar, 200 μm. (**D**) The number of IBA1^+^ cells after L4-SpN injury (day 7) in the ipsilateral or contralateral GN (*n* = 5 mice). **** *p*  <  0.0001, unpaired *t*-test. (**E**) Representative immunofluorescence images of IB4^+^ (yellow) and NF200^+^ (red) neurons in the L4-DRG (ipsilateral and contralateral sides) 14 days after injection of PBS, Ctrl-SAP, IB4-SAP, and CTB-SAP. Scale bar, 100 μm. (**F**) Quantification of the number of IB4^+^ or NF200^+^ DRG neurons (Nissl^+^) after injection of each saporin (Ctrl-SAP, IB4-SAP, and CTB-SAP) and PBS (*n* = 4–6 mice). Scale bar, 100 μm. * *p*  <  0.05 and ** *p*  <  0.01, one-way ANOVA with post hoc Tukey’s multiple-comparisons test. (**G**) Representative immunofluorescence images of IBA^+^ cells (green) in the SDH and GN after PBS and each saporin injection. Scale bars, 100 μm. Data are shown as mean  ±  SEM.

Given the minimal involvement of TRPV1^+^ nerve fibers in nerve injury-induced microgliosis in the SDH, the role of myelinated primary afferent fibers, such as Aβ-fibers, was investigated. Aβ-fibers from the lower spinal segments innervate not only the SDH but also the gracile nucleus (GN) in the brainstem [30]. Consistently, tdTomato^+^ fibers of L4-DRG neurons in the GN were observed (Figure 4A), most of which were ensheathed in MBP (Figure 4B), confirming the projection of myelinated primary afferent fibers from the L4-DRG. Furthermore, following L4-SpN injury in these mice, the number of IBA1^+^ cells increased in the GN, a spatial pattern closely matching the injured myelinated nerve fiber projection territories (Figure 4C,D). As the GN selectively receives primary afferent Aβ-fibers [30], these data suggest that primary afferent Aβ-fibers are involved in nerve injury-induced microgliosis.

To determine whether damage to primary afferent Aβ-fibers induces microgliosis, a pharmacological ablation approach with saporin-conjugated reagents was used. CTB-SAP was injected into the L4-SpN to target primary afferent Aβ-fibers, which express GM1 ganglioside, the target of CTB [20]. Intra-L4-SpN injection of CTB-SAP reduced the number of L4-DRG neurons expressing NF200 (a marker for large-diameter myelinated fibers, including Aβ-fibers) [31] compared with control saporin (Ctrl-SAP) (Figure 4E,F). Notably, CTB-SAP injection into the L4-SpN significantly increased IBA1 immunofluorescence and microglial cell number in the L4-SDH (Figure 4G). Similar microgliosis was also observed in the GN. However, Ctrl-SAP did not induce changes in microglia in the SDH, indicating that the microgliosis caused by CTB-SAP was not due to the non-specific effects of saporin itself. Additionally, intra-L4-SpN injection of IB4-SAP significantly decreased the number of IB4^+^ DRG neurons (Figure 4E,F) but did not cause microgliosis, as seen in CTB-SAP-treated mice (Figure 4G). Overall, these results demonstrate that pharmacological damage to primary afferent Aβ-fibers, but not C-fibers, is sufficient to induce microgliosis in the SDH.

## 4. Discussion

Since it was first reported in the late 1970s that microglia in the SDH respond to sciatic nerve axotomy [7,32], numerous studies have been published on the cellular and molecular alterations in SDH microglia following nerve injury [4,6]. However, the mechanism underlying the spatial heterogeneity of microgliosis in the SDH after nerve injury has not been fully elucidated. To shed light on this unresolved issue, this study developed a method for genetically labeling L4-DRG neurons by injecting an AAV into the L4-SpN and transecting the L4-SpN, thus specifically visualizing injured nerve fibers in the SDH and other regions. Using these nerve-labeled mice, we demonstrated for the first time that the spatial heterogeneity of nerve injury-induced microgliosis in the SDH is associated with the projection territories of injured myelinated primary afferent fibers, particularly Aβ-fibers. Indeed, reactive microglia after L4-SpN injury were preferentially localized along the projection pathways of myelinated fibers, including Aβ-fibers [33,34]. Additionally, microgliosis was observed in the GN and was highly restricted to the projection territory of injured myelinated fibers from L4-DRG neurons. Furthermore, pharmacological damage to NF200^+^ DRG neurons and their myelinated fibers using CTB-SAP was sufficient to induce microgliosis in both the SDH and the GN. Aδ-fibers and proprioceptor projections are also found in the GN [30], but, in contrast to Aβ-fibers, these projections in the GN were very limited. In addition, a spatial correlation between primary afferent fibers labeled with CTB and microgliosis resulting from peripheral nerve injury has been demonstrated in another model [14]. Therefore, Aβ-fibers would have a major contribution to nerve injury-induced microgliosis in the SDH and GN, although the possible involvement of other fibers (e.g., Aδ-fibers and proprioceptors) cannot be excluded.

In stark contrast, we found that unmyelinated C-fibers play a minimal role in microglial reaction. This differential role provides a crucial clue for elucidating the mechanisms underlying microgliosis after nerve injury. However, it should be noted that there are also contradictory findings regarding the role of unmyelinated C-fibers. Electrical stimulation of C-fibers in normal mice has been reported to cause microglia to become reactive in the SDH [35]. Additionally, it has recently been shown that conditional knockout of genes (GPR151 [36] and MyD88 [37]) in primary afferent C-fibers suppresses nerve injury-induced microgliosis in the SDH. In contrast, it has also been reported that nerve injury-induced microgliosis is not affected in rats whose TRPV1^+^ neurons have been ablated by systemic treatment with capsaicin during the neonatal period [38]. Although compensatory changes after the loss of TRPV1^+^ neurons could be considered, our study clearly demonstrated that acute denervation of TRPV1^+^ primary afferent fibers in the adult SDH does not influence SpN injury-induced SDH microgliosis, nor does the denervation itself cause microgliosis. These findings are supported by a previous study indicating that functional blockade of TRPV1^+^ C-fibers has no effect on nerve injury-induced microgliosis [39]. Moreover, pharmacological damage to IB4^+^ DRG neurons also did not result in microgliosis. Given that TRPV1^+^ and IB4^+^ fibers constitute most unmyelinated C-fibers [31], it appears that unmyelinated C-fibers are neither necessary nor sufficient to induce microgliosis in the SDH after peripheral nerve injury, emphasizing the significance of myelinated Aβ-fiber damage.

Several studies have reported the effect of selective gene loss in primary afferent neurons on nerve injury-induced microgliosis in the SDH [4,40]. Among these molecules, colony-stimulating factor 1 (CSF1) is currently considered the most promising candidate responsible for microgliosis following nerve injury [17,41]. CSF1 expression is rapidly induced in injured DRG neurons, and conditional knockout of CSF1 in primary afferent neurons significantly suppresses nerve injury-induced microgliosis in the SDH [17]. However, CSF1 induction is observed in injured DRG neurons with almost all soma sizes [41] (e.g., positive to NF200, CGRP, and IB4 [42]), suggesting that CSF1 upregulation occurs in various types of DRG neurons, including both myelinated Aβ-fibers and unmyelinated C-fibers [17,41], and that CSF1 in each DRG population may play a distinct role in nerve injury-induced microgliosis. Additionally, the role of demyelination of Aβ-fibers and its related factors (e.g., lysophosphatidic acid (LPA)) should also be considered [43,44,45,46]. Demyelination and LPA are known to induce microgliosis in various models of CNS disease [46,47]. To identify DRG neuron subtypes involved in microgliosis and to understand its molecular mechanisms (e.g., CSF1 and demyelination-related factors), further investigations using our established methods, which enable manipulation of gene expression in injured DRG neurons, are required.

It should also be noted that there are limitations to our methods. For example, intra-SpN injection of AAV vectors did not induce gene expression in all L4-DRG neurons, and the levels of gene expression varied among these neurons. This variability may explain why microgliosis in the SDH and the areas with tdTomato^+^ damaged myelinated fibers did not completely overlap. Technical improvements that enable gene expression in a larger number of DRG neurons are necessary for the future. Nevertheless, this is the first paper to establish a method, in conjunction with a neuropathic pain model developed by L4-SpN injury, that allows for gene expression specifically in injured primary afferent fibers. By incorporating genes used for optogenetics and chemogenetics, this technique could also be useful for functionally manipulating injured neurons and for elucidating the role of injured neurons in nerve injury-induced alterations in central nervous system function and pain behavior.

## 5. Conclusions

In this study, we established a novel method to visualize injured nerves using an AAV-mediated gene expression approach and demonstrated the spatial correlation between microgliosis and injured myelinated Aβ-fiber projections in the SDH and GN. Pharmacological damage to primary afferent Aβ-fibers was sufficient to induce microgliosis in both the SDH and the GN. Given that microgliosis in the SDH shortly after peripheral nerve injury is a critical step in the development of neuropathic pain [4], this study paves the way to identifying the molecular and cellular mechanisms underlying microgliosis following nerve injury and, consequently, to better understanding neuropathic pain.

## Data Availability

All data generated or analyzed during this study are included in the paper.

## Abbreviations

SDH	Spinal dorsal horn
L4	Fourth lumbar
DRG	Dorsal root ganglion
SpN	Spinal nerve
TRPV1	Transient receptor potential vanilloid 1
CNS	Central nervous system
IBA1	Ionized calcium-binding adapter molecule 1
AAV	Adeno-associated virus
ESYN	Enhanced synapsin
PBS	Phosphate-buffered saline
i.p.	Intraperitoneal
WT	Wild type
CTB	Cholera toxin B subunit
IB4	Isolectin B4
Ctrl	Control
SAP	Saporin
NF200	Neurofilament 200
MBP	Myelin basic protein
ROI	Region of interest
DIC	Differential interference contrast
n.s.	Not significant
GN	Gracile nucleus
CSF1	Colony-stimulating factor 1
LPA	Lysophosphatidic acid
